# Preparation and Formation Mechanism of Carbon Nanotubes via Coal Pyrolysis Using Alkaline Potassium Catalysts

**DOI:** 10.3390/nano15221691

**Published:** 2025-11-08

**Authors:** Tiankai Zhang, Qi Wang

**Affiliations:** Department of Basic Sciences, Shanxi Agricultural University, Taigu, Jinzhong 030801, China; zhangtiankai@sxau.edu.cn

**Keywords:** bituminous coal, catalytic pyrolysis, alkaline potassium, carbon nanotubes, growth mechanism

## Abstract

In this study, two potassium-based alkaline compounds, KOH and K_2_CO_3_, were utilized as catalysts for the pyrolysis of bituminous coal to directly synthesize carbon nanotubes (CNTs). During the conventional reaction process, CNTs were observed as unusual products, with respective contents reaching 9.43 and 8.98 wt.% in the pyrolysis products. The results indicate that at temperatures exceeding 600 °C, KOH undergoes complete conversion into K_2_CO_3_. The melting point of K_2_CO_3_ (890 °C) serves as the critical temperature for the formation of CNTs. Under the reducing atmosphere provided by –CH_2_– and aromatic C–C structures in coal, the transformation process K_2_CO_3_ → K_2_O → K occurs. At high temperatures, highly etching K reacts with the C–O–C structures in coal, converting them into carbon atoms and exposing the iron (Fe)-containing minerals in coal to the particle surface. Finally, the carbon atoms derived from the ether bonds form CNTs under the action of the native Fe catalyst in coal. The resulting product is a mixture of activated carbon and CNTs. This method fundamentally alters the composition of traditional coal-based activated carbon and provides a new approach for the cost-effective, large-scale production of high-value CNTs.

## 1. Introduction

Carbon nanotubes (CNTs) are renowned for their unique one-dimensional nanostructure and excellent physical and chemical properties, including extremely high mechanical strength [1], excellent electrical and thermal conductivity [2], and outstanding chemical stability [3]. These characteristics endow CNTs with tremendous application potential in various fields, such as electronic devices [4,5], energy storage [6], composite materials [7], and catalyst carriers [8]. Arc discharge and chemical vapor deposition (CVD) are two techniques commonly utilized to fabricate CNTs [9,10]. Arc discharge methods can be used to obtain CNTs with a high degree of graphitization at reaction temperatures of 3000–4000 °C [11,12]. Nonetheless, the harsh preparation conditions, complex equipment, and high energy consumption required for these methods lead to high operational costs. This has greatly limited the development of arc discharge technology and its utilization for the large-scale preparation of CNTs [9,11,12]. Although CVD can yield high-purity, high-quality CNTs at a relatively low cost, the lower preparation temperatures utilized in CVD processes result in a lower degree of graphitization and poor stability [13,14]. Therefore, the development of new, cost-effective, and environmentally friendly technologies for fabricating CNTs has significant scientific and industrial value [13,14].

Coal, as one of the most globally abundant fossil fuels, is traditionally used for power generation, metal processing, gasification, and chemical raw materials [15,16,17]. However, the existing applications of coal are inefficient and generate a large amount of greenhouse gases and pollutants, leading to certain adverse impacts on the climate, environmental stability, and human health [18,19,20,21]. Therefore, systematic exploration of high-value utilization methods for coal resources could help reduce environmental pollution, improve energy utilization efficiency, and achieve sustainable resource utilization [18,19,20,21]. One possible application for the high-value utilization of coal is the direct preparation of CNTs via pyrolysis. For instance, Pang et al. [22] were the first to propose the use of lignite to prepare CNTs, which could be generated with either straight or curved morphologies. Williams et al. prepared single-walled CNTs (SWCNTs) with a diameter of 1.2–1.7 nm from bituminous coal [23]. Qiu et al. [24] compared 11 types of bituminous coal for the preparation of CNTs, and concluded that using coal instead of graphite to prepare CNTs presents a strong economic advantage.

Arc discharge is the mainstream technology utilized to prepare CNTs from coal, and catalysts are often employed to promote the growth of CNTs. Wang et al. [25] filled graphite electrodes with copper(II) oxide (CuO) and anthracite for use in an arc discharge process, and obtained branched, heterogeneous CNTs with a diameter of about 40–60 nm. Qiu et al. [26] prepared double-walled CNTs with a diameter of 1–5 nm using anthracite, iron (Fe) powder, and coal tar as raw materials. Awasthi et al. [27] prepared SWCNTs with diameters of 1.7 and 1.2 nm using anthracite filled with Fe and nickel–yttrium (Ni–Y, 3:1) catalysts, respectively. Wilson et al. [28] analyzed the use of cobalt (Co) as a catalyst to prepare CNTs from raw coal (RC). Although the presence of Co promoted the production of CNTs, the low Co content in the RC suggested that CNTs were not present in the products unless additional Co was added. These studies demonstrate that transition metal elements such as Fe, Co, and Ni are the main catalysts that can promote the growth of CNTs via arc discharge.

Recently, studies have shown that alkaline catalysts, such as potassium hydroxide (KOH) and sodium hydroxide (NaOH), can effectively promote the growth of CNTs during coal pyrolysis [29,30]. When using these low-cost catalysts, the growth of CNTs can be controlled by adjusting the structure of coal and the pyrolysis conditions. Moreover, the use of alkaline K catalysts can reduce metal residues and improve the purity of CNTs. An additional benefit of utilizing alkaline K catalysts is that they are used to prepare CNTs via catalytic pyrolysis, which requires much lower reaction temperatures (<1000 °C) compared to arc discharge methods (3000–4000 °C) [9,29,31]. Therefore, the generation process of CNTs via catalytic pyrolysis is likely different compared to those generated via arc discharge. So far, only a few studies on the preparation of CNTs via alkaline K-catalyzed pyrolysis of coal have been reported [29,31]. Our research group previously reported a method for the direct preparation of CNTs via K-catalyzed coal pyrolysis and described the transformation of Fe in the coal during this process. This approach yielded a CNT and activated carbon composite that could be used as an electrode material without requiring further purification of CNTs. Compared with conventional activated-carbon electrodes, the electrodes fabricated from this composite exhibited markedly improved electrical conductivity, structural robustness, and ion-transport efficiency. Moreover, this composite demonstrated strong performance as a catalyst support and as an adsorbent material [32,33,34].

Alkaline catalysts play multiple roles in the formation process of CNTs. For example, Rashidi et al. [35] found that when magnesium oxide (MgO) was used as a catalyst support, its alkalinity possibly influenced the adsorption and decomposition behavior of carbon source gases (such as methane). This optimization of the carbon atom supply process favored the growth of CNTs. Li et al. [36] discovered Na-based catalysts (e.g., NaOH, Na_2_CO_3_) to be the efficient and low-cost catalysts for low-temperature growth of CNTs. Through a unique chemical catalytic pathway and a “self-cleaning” mechanism, they overcame the temperature limitations of traditional catalysts, enabling the growth of CNTs at low temperatures (as low as 450 °C). Modekwe et al. [37] observed that KOH, as an activating agent, significantly enhanced the yield and morphological controllability of Ni–Mo catalysts supported on corncob char for CNT production by the pyrolysis of waste polypropylene. This was achieved through the synergistic effects of “high-temperature pore creation, surface modification, and active phase regulation”.

Previous findings have indicated that during K-catalyzed coal pyrolysis, CNT growth is mainly promoted by the native Fe minerals in coal, such as siderite, and the addition of K elements promotes the following transformation process of Fe: FeCO_3_ → α-Fe → Fe_3_C [31]. However, key issues such as the temperature at which the formation of CNTs initiates, the mechanism of CNT generation, the form of the K elements before and after the formation of CNTs, and the precise roles of these K elements remain incompletely comprehended. Therefore, in this study, the generation temperature of CNTs; the changes in the form of K elements during the CNT formation process; and the changes in the functional groups, carbon structure, and crystal order of the coal were comprehensively evaluated. This study is expected to offer new insights into the growth mechanism of CNTs and provide a guiding reference for the high-value utilization of coal resources and the development of clean energy technologies.

## 2. Materials and Methods

### 2.1. Experimental Materials

First, Shaanxi bituminous coal (air-dried basis) was ground to below 74 μm to obtain the RC sample. The industrial analysis and elemental analysis results of the RC sample are listed in Table 1. The Fe content in the coal was 4.60 g·kg^−1^.

### 2.2. Catalyst Loading Method

First, KOH (15.0 g) was dissolved in a mixture of deionized water (30 mL) and ethanol (10 mL). Next, RC sample (30.0 g) was added to this solution, and magnetically stirred for 24 h. After stirring, this sample was dried in a forced-air-drying oven at 60 °C for 1 h and then at 105 °C for 5 h. Next, a mortar was used to evenly grind the dried sample (in block form). The ground sample was dried again at 105 °C for 2 h, then cooled down in a room-temperature desiccator for 30 min to obtain a coal sample loaded with KOH (denoted as RC-K). This catalyst loading process was also performed using K_2_CO_3_ (18.5 g, maintaining the same amount of K) instead of KOH, while keeping all other conditions constant to obtain a coal sample loaded with K_2_CO_3_ (denoted as RC-2K). Ethanol, KOH, and K_2_CO_3_ used in this procedure were all analytically pure and were purchased from Shanghai Aladdin Biochemical Technology Co., Ltd. (Shanghai, China).

### 2.3. Experimental Method

Pyrolysis was performed using 8.0 g of a coal sample (RC, RC-K, or RC-2K) placed in the constant-temperature middle zone of a horizontal tube furnace. Following sample loading, high-purity nitrogen gas was used to purge the air inside the reaction tube. The sample was heated to 600 °C at a rate of 10 °C·min^−1^ and held at this temperature for 90 min. Next, heating was turned off, and the tube furnace was allowed to cool down to room temperature to obtain the sample with a final pyrolysis temperature of 600 °C. Other samples were also pyrolyzed under final pyrolysis temperatures of 700, 800, and 900 °C.

The RC samples pyrolyzed at different temperatures were denoted as RC-X (where X represents the final pyrolysis temperature). After pyrolysis, the RC-K and RC-2K samples were washed with a suitable amount of dilute hydrochloric acid (1 M) to remove residual K, then repeatedly washed with distilled water until neutral. After washing, the samples were dried at 105 °C for 5 h, and the dried samples were denoted as RC-K-X or RC-2K-X. The sample nomenclature is summarized in Table 2.

### 2.4. Sample Characterization

Sample morphology was characterized by field-emission scanning electron microscopy (SEM, SU8020, Hitachi, Chiyoda City, Japan) with an acceleration voltage of 20 kV. The oxidation weight loss process of each sample was analyzed by thermogravimetric analysis (TGA, TA-60WS, Shimadzu, Kyoto, Japan) with a heating rate of 10 °C·min^−1^. The changes in the functional groups of each sample were analyzed via Fourier transform infrared (FTIR) spectroscopy (Nicolet iZ10, Thermo Scientific, Waltham, MA, USA) with a scanning range of 400–4000 cm^−1^ and a resolution of 0.4 cm^−1^. Quantitative analysis was performed using pellets prepared with 2.0 mg sample and 100.0 mg KBr. The crystal structure of each sample was determined by X-ray diffraction (XRD, Ultima IV, Rigaku, Tokyo, Japan) within the 2θ scanning range of 5–90° at a scanning speed of 4°·min^−1^ and a step size of 0.02°. The degree of defects in each sample was evaluated by Raman spectroscopy (RM2000, Renishaw, Gloucestershire, UK) with an excitation wavelength of 514 nm. The microstructure of the CNT walls and the elemental composition of the catalysts in the samples were analyzed by field-emission transmission electron microscopy (TEM, FEI Tecnai G2 F20, FEI Company, Hillsboro, OR, USA) together with energy-dispersive X-ray spectrometry (EDS, GENESIS, EDAX Inc., Mahwah, NJ, USA) operated using an accelerating voltage of 200 kV.

## 3. Results and Discussion

### 3.1. SEM Analysis

**Figure 1:**
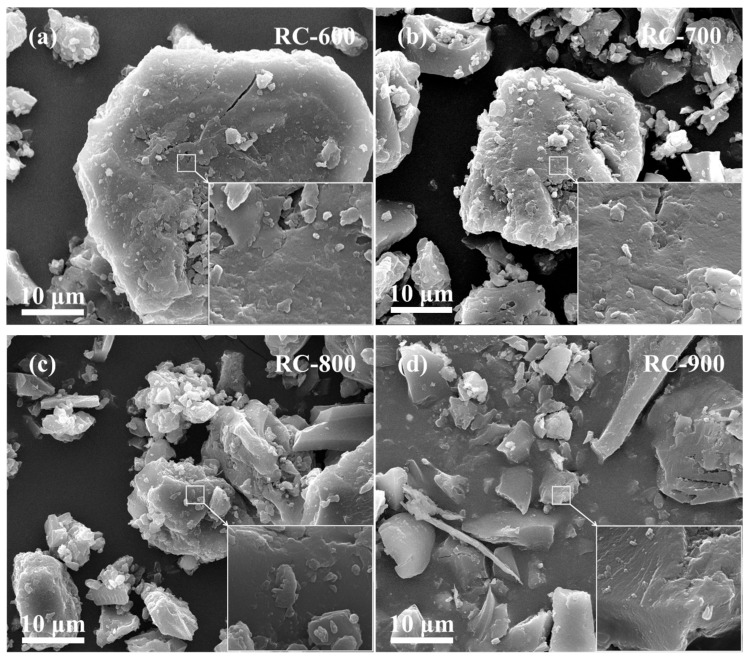

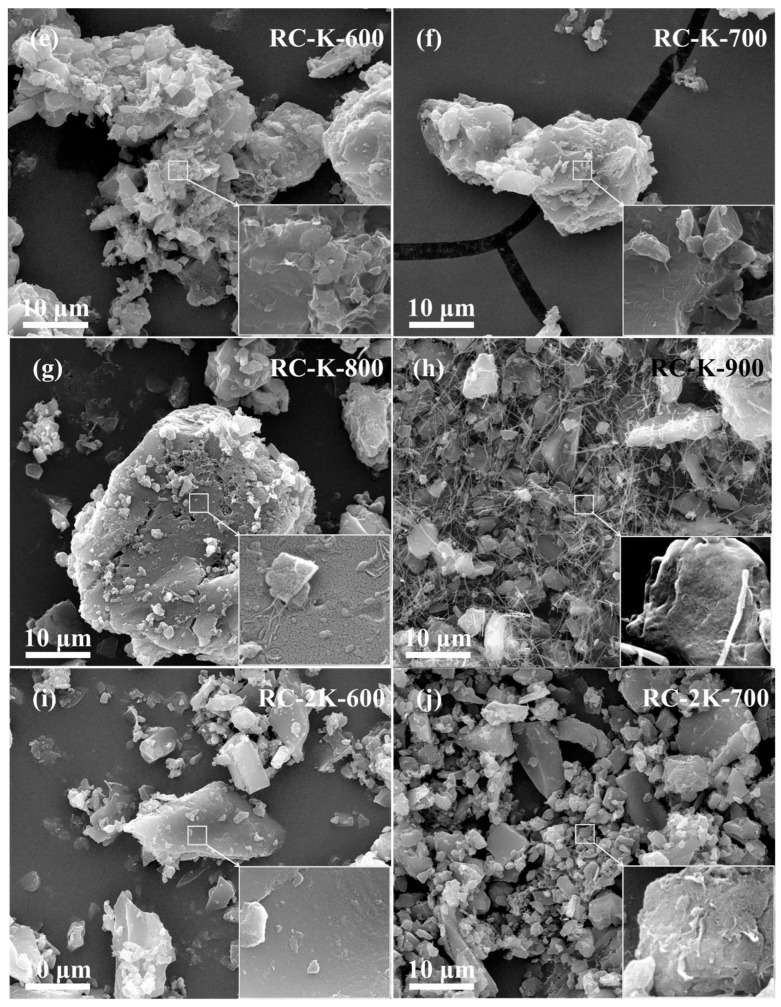

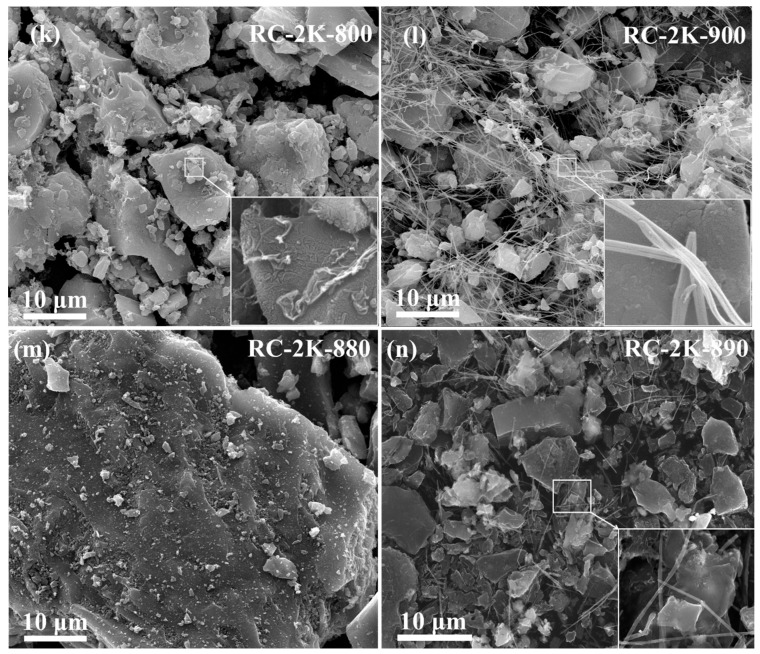
SEM images of (**a**–**d**) RC-X, (**e**–**h**) RC-K-X, and (**i**–**n**) RC-2K-X. (**m**,**n**) SEM analysis results of the pyrolysis products of RC-2K at 880 and 890 °C, respectively.

SEM images of the products obtained by the pyrolysis of RC, RC-K, and RC-2K samples at 600, 700, 800, and 900 °C are shown in Figure 1. The SEM images of RC-X (Figure 1a–d) show that with increasing pyrolysis temperature, the particle surface does not significantly change. The particle surfaces of RC-X are relatively flat, and no obvious porous structures are observed. The addition of KOH before pyrolysis (Figure 1e–h) leads to a certain etching phenomenon on the surface of RC-K during pyrolysis. With the increase in the pyrolysis temperature, this etching phenomenon gradually intensifies. After pyrolysis within the temperature range of 600–800 °C, no fibrous materials are observed on the surface of the solid RC-K-X particles. By contrast, the pyrolysis product RC-K-900 (pyrolyzed at 900 °C) shows the presence of numerous fibrous particles with a uniform morphology and lengths ranging from tens of micrometers. Replacing KOH with K_2_CO_3_ (Figure 1i–l) results in a relatively flat surface morphology after pyrolysis at 600 °C. Compared with RC-K-600, the surface of RC-2K-600 does not show any significant changes. However, when the pyrolysis temperature reaches 700 °C, etching phenomenon is observed on the RC-2K-700 surface, and with the further increase in the temperature, this etching phenomenon intensifies. Similarly to the RC-K-X samples, no fibrous materials are observed on the surface of RC-2K-X samples after pyrolysis within the temperature range of 600–800 °C. However, when the pyrolysis temperature rises to 900 °C, numerous fibrous particles can be observed. However, the fibrous particles of RC-2K-900 are somewhat longer than those of RC-K-900.

Comparative analysis of the SEM images of RC-X, RC-K-X, and RC-2K-X demonstrates that the addition of KOH or K_2_CO_3_ leads to the enhanced generation of small-sized solid particles. Based on the analysis reported in previous studies, the fibrous materials observed in RC-K-900 and RC-2K-900 are multi-walled CNTs [29,31]. Overall, the SEM analysis demonstrates that the addition of KOH and K_2_CO_3_ provides a certain etching effect on the raw coal, and both K catalysts promote the generation of CNTs. However, both catalysts require a higher pyrolysis temperature (~900 °C) to exhibit this effect.

### 3.2. TG-DTG Analysis

**Figure 2:**
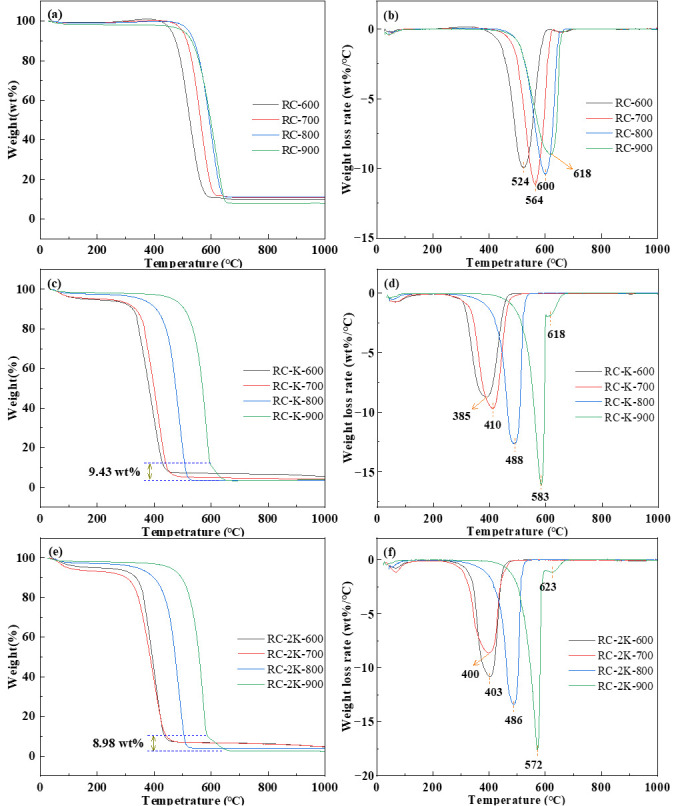
TG-DTG analysis of samples: (**a**) RC-TG, (**b**) RC-DTG, (**c**) RC-K-TG, (**d**) RC-K-DTG, (**e**) RC-2K-TG, and (**f**) RC-2K-DTG.

The TGA results of the RC-X, RC-K-X, and RC-2K-X samples are shown in Figure 2. The RC-X samples undergo maximum weight loss of about 90 wt.%, and each differential thermogravimetric (DTG) curve shows a main weight loss peak as well as a smaller peak at around 670 °C (Figure 2a,b). The temperatures corresponding to the maximum weight loss rate of RC-600, RC-700, RC-800, and RC-900 are 524, 564, 600, and 618 °C, respectively. This suggests that as the pyrolysis temperature increases, the carbon structure of the pyrolysis products gradually becomes more ordered, which is consistent with existing research results [38]. The weight loss peak near 670 °C may be attributed to the calcium carbonate component in the coal (as described in Section 3.4) [39,40].

The maximum weight loss of RC-K-X gradually increases from 94.37 to 96.48 wt.% with increasing pyrolysis temperatures (Figure 2c,d). These samples show slightly higher maximum weight losses compared to the RC-X samples. The temperatures corresponding to the maximum weight loss rates of RC-K-600, RC-K-700, RC-K-800, and RC-K-900 are 385, 410, 488, and 583 °C, respectively, showing an upward trend. However, these maximum weight loss temperatures are lower than those of the RC-X samples pyrolyzed at the same temperature. This result indicates that the addition of KOH disrupts the carbon structure of the raw coal and increases the oxidation activity of the solid products to a certain extent [41]. Notably, the DTG curves of RC-K-600, RC-K-700, and RC-K-800 contain only one weight loss peak, while that of RC-K-900 shows two weight loss peaks. This can be ascribed to the presence or absence of CNTs. The SEM images of RC-K-600, RC-K-700, and RC-K-800 show the presence of only one type of particle, with no CNT generation, while that of RC-K-900 shows two types of particles. Therefore, the weight loss peak near 618 °C can be attributed to CNTs. However, the proportion of CNTs in RC-2K-900 is relatively small (corresponding to a CNT content of about 9.43 wt.%).

The weight loss and DTG curves of RC-2K-X (Figure 2e,f) are basically consistent with those of RC-K-X. The content of CNTs in RC-2K-900 is about 8.98 wt.%. Comparative analysis of the DTG curves of RC-K-X and RC-2K-X shows that for pyrolysis temperatures above 600 °C, the temperature corresponding to the maximum weight loss rate of RC-K-X is slightly higher than that of RC-2K-X. Therefore, although KOH is more strongly alkaline than K_2_CO_3_, the destructive effect of KOH on the ordered structure of the coal is weaker than that of K_2_CO_3_. This is potentially due to the fact that KOH reacts more extensively with the oxygen-containing functional groups present in the coal [42]. This would lead to the formation of more stable chemical structures, which would require higher temperatures to undergo oxidation and decomposition. Moreover, it is also possible that KOH promotes the transformation of active groups in coal (such as –CH_3_, –COOH, etc.) to less active structures. Consequently, the proportion of relatively less active large molecular carbon structures gradually increases, indicating that oxidation and decomposition need to be carried out at higher temperatures [43].

Notably, although the same content of K was added to both RC-K and RC-2K, the CNT content in RC-K-900 was higher than that in RC-2K-900. This suggests that KOH is more conducive to the production of CNTs than K_2_CO_3_, which is potentially due to the stronger alkalinity of KOH compared to K_2_CO_3_. During the coal pyrolysis process, KOH reacts with some functional groups in the coal, etching the surface structure of the coal particles while exposing more of the metal elements (such as Fe) within the coal. Previous studies have shown that the exposure of Fe-containing minerals present in the coal structure via etching plays an important role in the production of CNTs [31]. Another reason may be that within the temperature range of 400–700 °C, KOH can react with some carbon-containing functional groups in the raw coal, leading to its transformation into K_2_CO_3_. Even at a low temperature of 400 °C, KOH has been reported to be completely transformed into K_2_CO_3_. This is similar to the results of the present study. In this study, the XRD analysis presented in Section 3.4 confirms that a pyrolysis temperature of 600 °C is sufficient for the complete conversion of KOH into K_2_CO_3_ [44]. Another potential reason explaining the enhanced CNT production performance of KOH is the gradual reaction of KOH with some functional groups to form porous structures in the coal during pyrolysis [45]. Under high-temperature conditions (900 ℃), these porous structures facilitate the movement and intercalation of K_2_O and K, promoting the generation of carbon source required for the growth of CNTs. However, at temperatures below 900 °C, these carbon-containing functional groups that can react with KOH are not likely to provide the carbon source required for the growth of CNTs.

### 3.3. FTIR Spectroscopy Analysis

**Figure 3:**
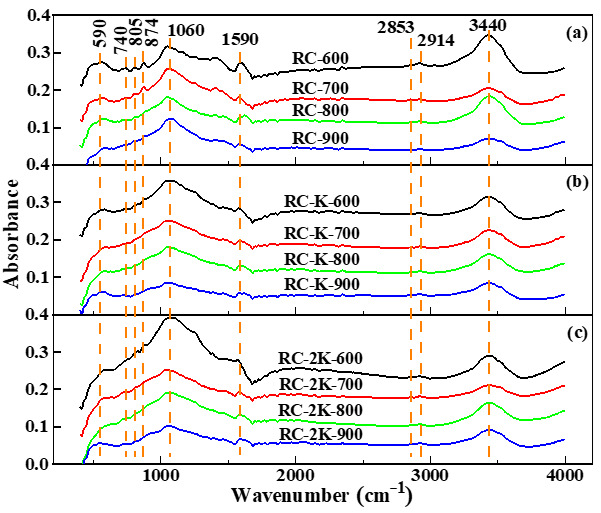
FTIR spectra of (**a**) RC-X, (**b**) RC-K-X, and (**c**) RC-2K-X.

The RC-X, RC-K-X, and RC-2K-X pyrolysis products exhibit similar FTIR spectra, as shown in Figure 3. Absorption peaks are observed at 590, 1060, 1590, 2853, 2914, and 3440 cm^−1^, and the functional groups corresponding to these absorption peaks are listed in Table 3. The absorption peak of the samples near 3440 cm^−1^ is ascribed to the stretching vibration of –OH, which generally exists in the form of intermolecular hydrogen bonds, free –OH, and terminal –OH groups in the aromatic structure of coal [46]. Two relatively weak absorption peaks near 2914 and 2853 cm^−1^ are ascribed to the asymmetric stretching vibration of aliphatic –CH_2_ and the symmetric stretching vibration of aliphatic –CH_3_, respectively. The absorption peak intensity at 2914 cm^−1^ is greater than that at 2853 cm^−1^, indicating that the –CH_2_ in the samples exists in the form of relatively long aliphatic side chains [29,47]. The absorption peak near 590 cm^−1^ represents the stretching vibration of Si–O and Si–O–Al, indicating the existence of Si- and Al-containing minerals. This is consistent with the results of previous studies [29,31].

The FTIR spectra of RC-600 and RC-700 (Figure 3a) exhibit some weak absorption peaks near 874, 805, and 740 cm^−1^, indicating the stretching vibration of aromatic C–H. However, in the FTIR spectra of RC-800, RC-900, RC-K-X, and RC-2K-X samples (Figure 3b,c), the peaks corresponding to these functional groups gradually disappear. This indicates that with the increase in the pyrolysis temperature and in the presence of KOH or K_2_CO_3_, the condensation reactions of the coal gradually become more intense and the large molecular structure of the coal becomes more ordered, which is consistent with the TG-DTG analysis. Furthermore, with increasing pyrolysis temperature, the intensity of the absorption peak at 1060 cm^−1^ in the FTIR spectra of RC-X (corresponding to aromatic C–C bonds, C–H deformation vibration, and C–O–C ether bond stretching vibration) gradually increases. This indicates that the pyrolysis of RC promotes the generation or accumulation of C–C and C–H aromatic carbon bonds as well as C–O–C ether bonds. Conversely, the intensity of the same peak in the FTIR spectra of RC-K-X and RC-2K-X gradually decreases with increasing pyrolysis temperature. Therefore, the addition of KOH or K_2_CO_3_ is not conducive to the generation or accumulation of these groups. This is because KOH added in the early stage can transform into K_2_CO_3_ by reacting with the carbon framework in the coal. Further, the as-produced K_2_CO_3_ reacts with CO and H_2_ produced during the coal pyrolysis process as well as the carbon framework in the coal at high temperatures to generate K_2_O, K, and other K-containing substances. These K-containing substances exhibit strong etching properties and intercalation capabilities at high temperatures, disrupting the original carbon structure in the coal [55].

The FTIR spectroscopy analysis demonstrates that aromatic C–C, C–H, and C–O–C ether bond structures are the main functional groups affected by the pyrolysis of RC, RC-K, and RC-2K. Notably, CNTs are not formed at pyrolysis temperatures of 800 °C or below, with CNT production only observed after pyrolysis at 900 °C. This demonstrates that the added KOH and K_2_CO_3_ catalysts act at high temperatures. At high temperatures, KOH converts to K_2_CO_3_, which in turn influences the production of CNTs due to its high-temperature properties. Previous studies have shown that the main catalysts for the growth of CNTs during coal pyrolysis are the native Fe-containing minerals distributed inside the coal particles. However, surface etching is necessary to expose the Fe atoms to the surface of the coal particles, where they can catalyze the production of CNTs. Therefore, the observation in this study that CNTs are only produced at 900 °C is potentially due to the stronger etching ability of K_2_CO_3_ at this temperature. SEM analysis of the products generated at slightly lower temperatures (Figure 1m) shows that CNTs are not produced at a pyrolysis temperature of 880 °C, but a small amount of CNTs appear after pyrolysis at 890 °C (Figure 1n). Comparison with the CNT content generated by pyrolysis at 900 °C (Figure 1l) indicates that CNT generation begins between 890 and 900 °C. This may be attributed to the enhanced fluidity of K_2_CO_3_ when its melting point (890 °C) is surpassed along with the decomposition of some of the K_2_CO_3_ [56]. The K_2_O produced during this decomposition process reacts with C in the coal to produce K. The K atoms can strongly intercalate into the coal [44], where they further etch the carbon structure of the coal to expose more Fe-containing minerals. Below its melting point, K_2_CO_3_ maintains its original state, with relatively low fluidity and chemical activity [56]. Therefore, K_2_CO_3_ has a limited etching effect on the carbon structure in the coal at pyrolysis temperatures below 890 °C, and thus CNTs do not appear in the products.

### 3.4. XRD Analysis

**Figure 4:**
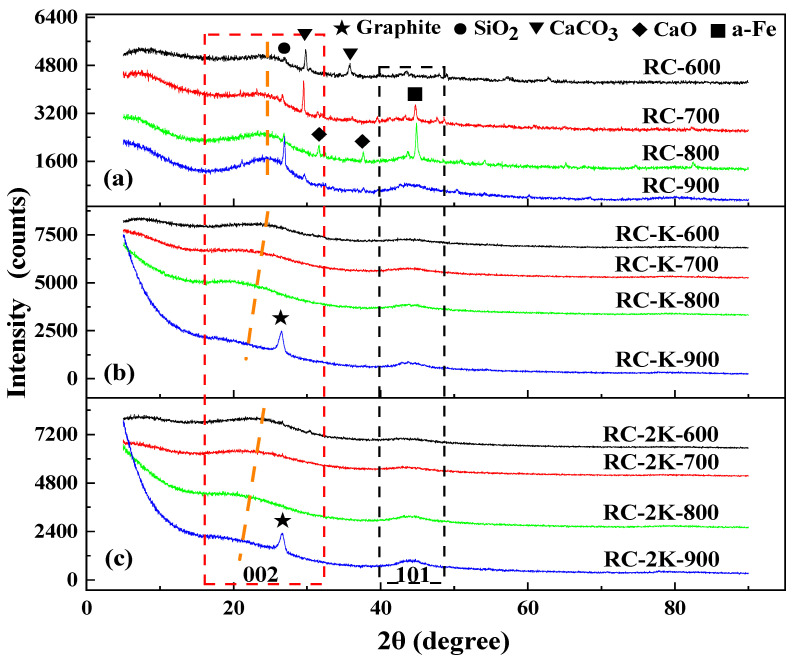
XRD patterns of (**a**) RC-X, (**b**) RC-K-X, and (**c**) RC-2K-X.

The pyrolyzed samples were analyzed by XRD, as depicted in Figure 4. Samples RC-600 and RC-700 exhibit diffraction peaks associated with CaCO_3_ (Figure 4a), indicating the presence of calcite in the raw coal. However, in the XRD pattern of RC-800, the diffraction peaks of CaCO_3_ disappear, and diffraction peaks ascribed to CaO are observed instead [57]. This indicates that calcite decomposes when the coal is pyrolyzed at 800 °C. Moreover, the diffraction patterns of RC-700 and RC-800 show a diffraction peak corresponding to α-Fe, which is ascribed to the decomposition and transformation of siderite in the raw coal during the pyrolysis process. This is consistent with the results of a related previous study [31]. A diffraction peak of silica (SiO_2_) is located near 26.9° in the diffraction patterns of all the RC-X samples, which is due to the presence of a certain amount of quartz in the raw coal. With the increase in the pyrolysis temperature, this SiO_2_ peak becomes more intense, indicating the increase in the relative content of quartz in the pyrolyzed samples. The diffraction peaks present in the diffraction patterns of RC-X are not observed in the diffraction patterns of RC-K-X and RC-2K-X (Figure 4b,c). This is because the KOH or K_2_CO_3_ added to these samples reacts with the mineral components in the coal at high temperatures, which are removed during the subsequent acid washing process.

Comparative analysis of Figure 4b,c reveals that at the same temperature, the XRD patterns for the RC-K-X and RC-2K-X samples exhibit similar morphologies. When the temperature increases from 600 to 900 °C, the 002-diffraction peak of amorphous carbon (denoted by the red dotted rectangle) gradually shifts toward lower angles (as shown by the orange dotted line in the figure), while the corresponding full width at half maximum (FWHM) gradually increases. This result indicates a gradual increase in the amorphous carbon content within the samples, which is attributed to the etching effect of the K component at high temperatures, leading to the transformation of the originally ordered carbon structures in coal into amorphous carbon structures. Furthermore, the higher the temperature, the stronger the etching capability of the K component. Among twelve samples, only RC-K-900 and RC-2K-900 exhibit the diffraction peak corresponding to graphite (002), at approximately 26.6°. However, compared to the sharp peak characteristic of pure graphite, the diffraction peaks of these two samples broaden with an increased FWHM, indicating that although CNTs are present, the crystalline order of the obtained CNTs is lower than that of pure graphite.

**Figure 5:**
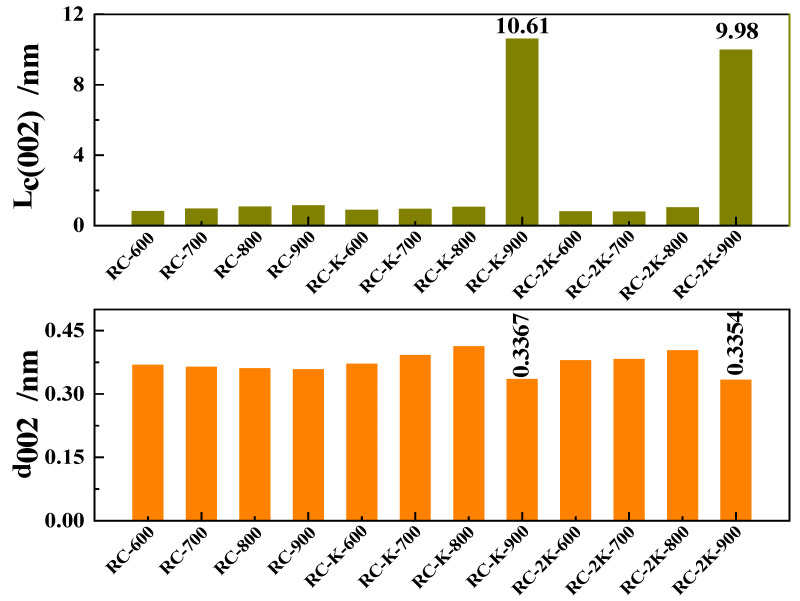
The stacking height Lc (002) and interlayer spacing d_002_ of the samples RC, RC-K, and RC-2K.

Next, the 002 peaks of the samples were fitted by using the Gaussian functions, and the lattice parameters of the samples, namely, stacking height (Lc (002)) and interlayer spacing (d002) of the graphitic crystallites, were obtained by using the Scherrer equation and Bragg’s law, respectively [58]. Figure 5 shows the corresponding results, revealing that with increasing temperature (within the range of 600–900 °C), the Lc (002) of RC-600, RC-700, RC-800, and RC-900 slowly increases to approximately 1 nm, while the d_002_ slowly decreases to approximately 0.36 nm. The primary reason for the decrease in d_002_ across the samples is likely the continuous release of volatiles from the coal during pyrolysis, leading to a gradual decrease in the interlayer spacing of the residual solid carbon structures. This is simultaneously accompanied by condensation reactions, which leads to the gradual increase in the Lc (002) of the remaining carbon structures.

After adding KOH to the raw coal, and increasing the pyrolysis temperature within the range of 600–800 °C, the Lc (002) and d_002_ of RC-K-600, RC-K-700, and RC-K-800 gradually increase, but the magnitude of change is relatively small. When the temperature rises to 900 °C, the Lc (002) increases rapidly to approximately 10.0 nm, while the d_002_ decreases to approximately 0.33 nm, close to the theoretical d_002_ value of graphite (0.3354 nm). The changes in Lc (002) and d_002_ for RC-2K-600, RC-2K-700, RC-2K-800, and RC-2K-900 follow the same trend as described above. The primary reason for the abrupt changes in Lc (002) and d_002_ with increasing temperature from 800 to 900 °C is the formation of CNTs in the product. In the presence of KOH or K_2_CO_3_, the etching ability of the K atoms gradually strengthens with increasing temperature within the range of 600–800 °C, which disrupts the original relatively ordered carbon structure and increases the interlayer spacing. However, when the pyrolysis temperature rises to 900 °C, the generation of CNTs dominates compared to the etching action of the K atoms. Consequently, the d_002_ decreases and the L_c_ (002) value sharply increases.

Figure 4 illustrates that all three sample types of RC, RC-K, and RC-2K exhibit a diffraction peak (as indicated by the black dotted rectangle) at around 44°, which correspond to the (101) plane of graphitic crystallite carbon structures. Within the temperature range of 600–900 °C, as the pyrolysis temperature increases, the intensity of the (101) diffraction peak gradually strengthens and its FWHM gradually decreases for the raw coal, RC-K, and RC-2K samples, indicating that the peak shape becomes progressively sharper. This trend suggests that increase in the pyrolysis temperature facilitates the extension of aromatic structures in coal, ultimately leading to a gradual increase in the content of graphitic crystalline structures [59]. This observed change also implies that the aromatic carbon structures in coal are unlikely to be the primary carbon source for the growth of CNTs. Instead, the carbon required for CNT formation is mainly supplied by aliphatic groups, ether bonds, or other reactive carbon-containing structures.

**Figure 6:**
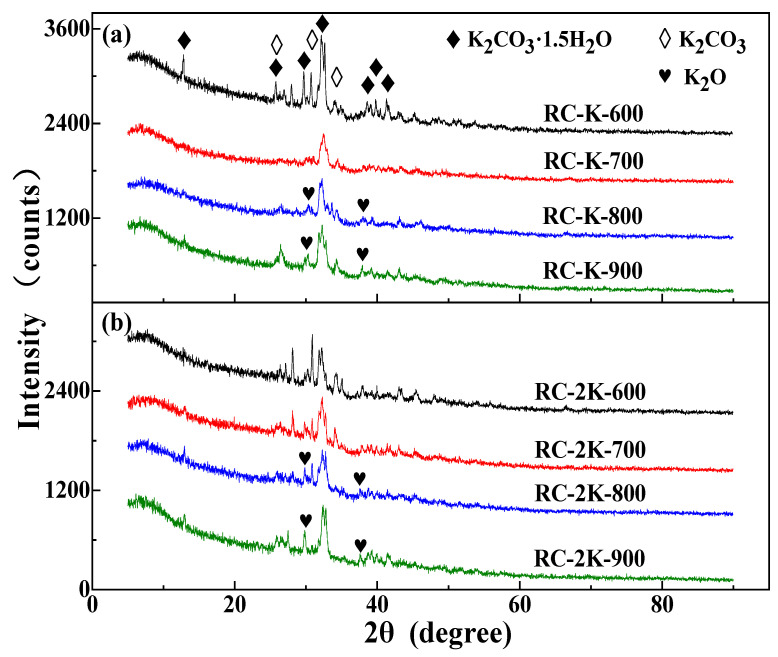
XRD patterns of the samples without acid washing treatment: (**a**) RC-K-X and (**b**) RC-2K-X.

Further, RC-K-X and RC-2K-X samples were also evaluated by XRD prior to acid treatment, as shown in Figure 6. The KOH and K_2_CO_3_ added to these samples mainly exist in the form of K_2_CO_3_. This demonstrates that during pyrolysis, the KOH first transforms into K_2_CO_3_, and the K_2_CO_3_ then etches the large molecular carbon structures in the coal at high temperatures to provide a carbon source for the growth of CNTs. K_2_O peaks are observed in the diffraction patterns of RC-K-900 and RC-2K-900, which is ascribed to the decomposition of K_2_CO_3_ into K_2_O at high temperatures. This K_2_O can react with H_2_ generated by the molecular condensation reactions within the coal under the high-temperature conditions, leading to their reduction into K atoms. The K_2_O and K components exhibit strong etching and intercalation properties, and together with K_2_CO_3_, they etch the solid carbon structure in the coal to provide a carbon source for the growth of CNTs.

### 3.5. Raman Spectroscopy Analysis

**Figure 7:**
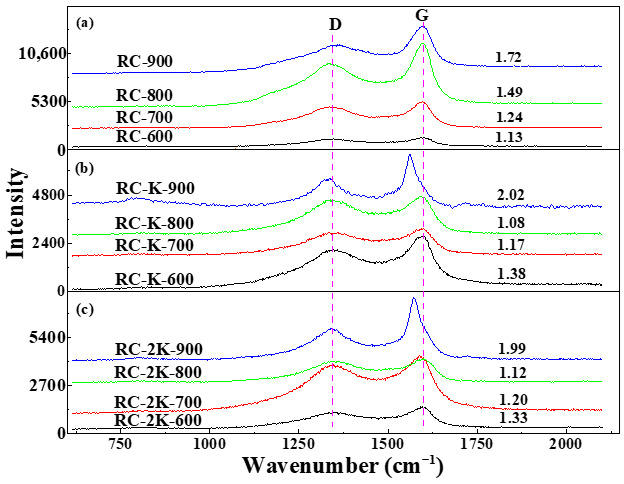
Raman spectra of (**a**) RC-X, (**b**) RC-K-X, and (**c**) RC-2K-X.

The Raman spectra of RC-X, RC-K-X, and RC-2K-X are shown in Figure 7. These first-order Raman spectra show two bands, and the D band near ~1340 cm^−1^ corresponds to the disordered model A_1g_. The intensity of this band reflects the degree of disorder of the carbon structure in each sample (i.e., the presence of defect structures caused by carbon atom vacancies or heteroatom substitutions) [29]. The G band near ~1590 cm^−1^ represents the E_2g_ stretching vibration mode of the aromatic layers in the graphite crystal structure, and the strength of this band is related to the degree of order of the graphite structure in each sample [60]. The intensity ratio of the G band to the D band (I_G_/I_D_) reflects the degree of graphitization or the degree of order of the internal carbon structure. A lower I_G_/I_D_ ratio indicates a less ordered carbon structure and a lower degree of graphitization. Conversely, a higher I_G_/I_D_ ratio indicates a more ordered carbon structure and a higher degree of graphitization.

Figure 7a shows the Raman spectra of the raw coal during individual pyrolysis, which exhibit typical thermally driven graphitization characteristics. With the increase in the temperature from 600 to 900 °C, the I_G_/I_D_ ratios of the samples RC-600, RC-700, RC-800, and RC-900 are 1.13, 1.24, 1.49, and 1.72, respectively, showing an overall upward trend. Concurrently, the intensity of the G-band gradually increases. This indicates continuous expansion in the size of sp^2^ carbon microcrystals in the samples, and improvement in the structural ordering. The stable position of the G-band at approximately 1590 cm^−1^ suggests that no significant stress or doping was introduced during this process.

Figure 7b,c demonstrate that after the addition of KOH or K_2_CO_3_, the Raman analysis results of the samples exhibit more complex behavior. Within the temperature range of 600–800 °C, the I_G_/I_D_ ratio gradually decreases with increasing pyrolysis temperature, which is opposite to the trend observed for the directly pyrolyzed raw coal samples. The reason for this lies in the role of KOH as an “etching agent.” At this stage, KOH preferentially attacks and gasifies the disordered carbon phases generated during coal pyrolysis. This process disrupts the initially formed small ordered regions, leading to an enhancement in the relative Raman signal of defects, thereby manifested as a decrease in the I_G_/I_D_ ratio. When the temperature reaches the threshold of 900 °C, the I_G_/I_D_ ratio increases significantly, and the G-band exhibits a red shift. This marks the transition of the role of KOH from an “etching agent” to a “high-efficiency catalyst” and “dopant”. At this point, the KOH catalytic system is fully activated, driving the intrinsic growth of high-quality, low-defect CNTs (resulting in an increase in I_G_/I_D_). Simultaneously, the K species generated at high temperatures effectively perform n-type doping (and possibly introduce compressive stress) on the newly formed CNTs, softening the carbon–carbon bonds and thus causing the red shift in the G-band [61].

The analysis of Raman data reveals the unique temperature-dependent transition in the catalytic role of alkaline K catalysts. Below 900 °C, they primarily modify the carbon structure through etching; above this temperature, they act as powerful catalysts for the direct synthesis of highly crystalline and dopable CNTs.

### 3.6. TEM-EDS Analysis

**Figure 8:**
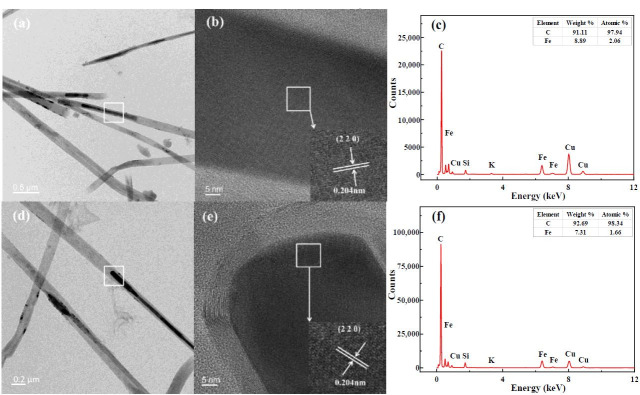
TEM images of (**a**) RC-K-900 and (**d**) RC-2K-900, magnified views of the white boxed areas in (**b**) a and (**e**) d, and EDS analysis results of the white boxed areas in (**c**) b and (**f**) e.

In addition to the conventional elements C, H, O, N, and S, coal typically contains impurity elements such as Fe, Si, Al, Na, and Ni. Among these, Fe and Ni are commonly used as catalysts in existing methods for synthesizing CNTs. To investigate the elemental composition of the catalysts required for CNT growth during K-catalyzed coal pyrolysis, RC-K-900 and RC-2K-900 were analyzed by TEM-EDS, as shown in Figure 8.

Figure 8a illustrates that the CNTs in RC-K-900 exhibit a straight morphology with diameters of approximately 100–200 nm, and some CNTs encapsulate the catalyst particles. Figure 8c demonstrates that the catalyst particles in this catalyst primarily consist of C and Fe, with trace amounts of Si, K, and Cu. Notably, C, Fe, and Si originate from the coal itself, K comes from the externally added KOH, and Cu originates from the copper support grid used during analysis. Figure 8b shows that the catalyst particles possess a lattice spacing of approximately 0.204 nm, corresponding to the (220) crystal plane of Fe_3_C [62,63]. Furthermore, previous research findings indicate that when FeCO_3_ was used to simulate the Fe species in coal during alkaline K-catalyzed coal pyrolysis for CNT production, the Fe ultimately existed in the form of Fe_3_C [31]. This alignment between the two research results demonstrates that the inherent Fe present in the coal facilitates the formation of CNTs during the pyrolysis of coal. The TEM and EDS results of RC-2K-900 align closely with those of RC-K-900. Collectively, these analyses demonstrate that CNT formation is catalyzed by Fe present in coal during alkaline K-catalyzed coal pyrolysis.

**Figure 9:**
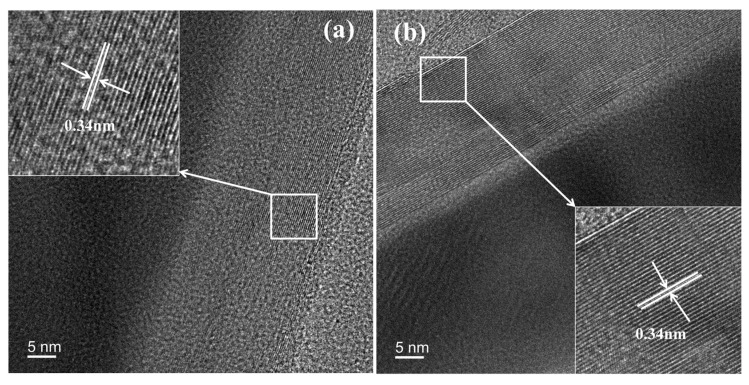
TEM images of (**a**) RC-K-900 and (**b**) RC-2K-900.

Figure 9 shows that the CNTs in RC-K-900 exhibit 58 wall layers, while those in RC-2K-900 possess 61 wall layers. The interlayer spacing of the CNTs in both products measures approximately 0.34 nm, corresponding to the lattice parameter of the graphite (002) plane. These results demonstrate that the CNTs synthesized through alkaline K-catalyzed coal pyrolysis feature a highly ordered crystalline structure with minimal defects.

### 3.7. Mechanism of Carbon Nanotube Growth

When the KOH-catalyzed coal was pyrolyzed within the temperature range of 600–900 ℃, the added KOH existed in the form of K_2_CO_3_. This is mainly due to the reaction of KOH with side chains in the coal (such as ethyl groups) leading to its transformation into K_2_CO_3_ and K_2_O, as shown in Equation (1).

(1) 4KOH + –CH_2_– → K_2_CO_3_ + K_2_O + 3H_2_

Simultaneously, during the heating process, KOH may undergo decomposition, as shown in Equation (2) [64].

(2) 2KOH → K_2_O + H_2_O↑

When the RC is pyrolyzed in the absence of a K catalyst, graphite is generated but CNTs are not produced at 900 °C. However, some areas on the surface of graphite are significantly etched [31]. This indicates that the elemental carbon in the aromatic structure of the pyrolyzed coal does not act as a carbon source for the growth of CNTs. Instead, in the alkaline K-catalyzed samples, this aromatic carbon mainly plays a reducing role in transforming the K species. FTIR spectroscopic analysis shows that the content of aromatic C–C in the K-catalyzed samples gradually decreases during the pyrolysis process. This is mainly because K_2_CO_3_ and K_2_O derived from KOH (or the directly added K_2_CO_3_) react with the aromatic C–C in the coal, as shown in Equations (3) and (4).

(3) 2K_2_CO_3_ + C–C → 2K_2_O + 4CO↑

(4) 2K_2_O + C–C → 4K + 2CO↑

The addition of either KOH or K_2_CO_3_ leads to the formation of CNTs during coal pyrolysis at 900 °C. However, CNTs are not produced at pyrolysis temperatures below 890 °C. This is mainly because K_2_CO_3_ is in a molten state and undergoes decomposition only above 890 ℃, as shown in Equation (5) [65]. The molten state of K_2_CO_3_ is a key factor in catalyzing the generation of CNTs.

(5) $$K_{2}{\mathrm{CO}}_{3}\;⇌\;K_{2}O\;+\;CO_{2}$$

Notably, K shows strong etching properties at high temperatures. Moreover, the aromatic C–C, C–H, and C–O–C ether bond groups in the coal are still undergoing transformations at temperatures above 600 °C (Figure 3). Therefore, Equations (6)–(10) [66] represent the key steps in the production of CNTs during the K-catalyzed coal pyrolysis process. That is, the aromatic C–C, C–H, and ether bond C–O–C groups are transformed into the carbon source that is utilized to grow the CNTs under the action of K and K_2_O. At the same time, as the reaction proceeds, Fe particles inside the coal particles are exposed to the surface due to etching, where they act as catalysts and catalyze the production of CNTs [31].

(6) K_2_O + C–C → 2K + CO↑ + C

(7) 2K_2_O + 2C-H → 4K + 2CO↑ + H_2_↑

(8) 2K + C–O–C → K_2_O + 2C

(9) $$\mathrm{Fe}\;(\mathrm{inside}\;\mathrm{the}\;\mathrm{coal}\;\mathrm{particle})\;\overset{K,\;K_{2}O}{\to }\;\mathrm{Fe}\;(\mathrm{surface}\;\mathrm{of}\;\mathrm{the}\;\mathrm{coal}\;\mathrm{particle})$$

(10) $$C+C+C\;\overset{Fe}{\to }\;\mathrm{CNTs}$$

After the completion of reaction, the K_2_O generated during this process reacts with carbon dioxide (CO_2_) generated during coal pyrolysis, leading to its conversion into K_2_CO_3_, as shown in Equation (11). During the cooling or sample detection process, some of the K_2_CO_3_ absorbs water to form K_2_CO_3_·1.5H_2_O.

(11) K_2_O + CO_2_ → K_2_CO_3_

Compared with K_2_CO_3_, the transformation process of KOH is relatively complex, as shown in Equations (1) and (2). These steps are conducive to enriching the internal pore structure of the coal particles, which is beneficial for the transformation of the carbon source required for the growth of CNTs. This also promotes the exposure of Fe. Owing to these differences, the KOH-catalyzed pyrolysis reaction provides greater CNT content compared to K_2_CO_3_-based catalysis.

Compared to conventional applications of KOH or K_2_CO_3_, such as production of activated carbon through coal pyrolysis (primarily utilizing the etching effect of K species) or assisting hydrocarbon gas pyrolysis for synthesizing CNTs (mainly serving as catalyst promoters), the direct synthesis of CNTs via alkaline K-catalyzed coal pyrolysis demonstrates a dual role of KOH or K_2_CO_3_. First, prior to CNT formation, they primarily supply K_2_O and metallic K to the system. Second, they expose the Fe-containing minerals within the coal particles to the surface. The primary functions of K_2_O and metallic K are to extract carbon atoms from functional groups in coal, such as C–C, C–H, and ether bonds (C–O–C), and simultaneously facilitate the migration of Fe-containing minerals to the particle surface. The Fe atoms in the coal primarily function to adsorb carbon atoms or carbon-containing units and convert them into CNTs.

**Figure 10:**
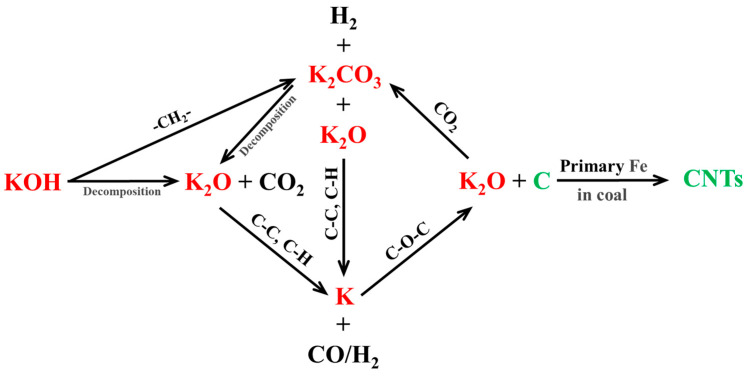
Schematic illustration of K element transfer and CNTs formation. The red text indicates the transformation process of potassium (K), the black text represents the functional groups or substances involved in the reaction, and the green text denotes the formation process of CNTs.

In the alkaline K-catalyzed coal pyrolysis system, the formation mechanism of CNTs is as follows: First, during the heating process (600–900 °C), KOH or K_2_CO_3_ reacts with carbon-containing groups in the coal, such as –CH_2_– and aromatic C–C, or undergoes decomposition, generating K_2_O and metallic K. Subsequently, at high temperatures (~900 °C), K_2_O and metallic K react with groups such as C–C, C–H, and ether bonds (C–O–C) in the coal, releasing carbon atoms from these groups. Concurrently, through an “etching effect” on the coal particles, the Fe atoms within the coal get exposed from the interior to the surface. Finally, these carbon atoms or carbon-containing units, derived from groups such as C–C, C–H, and ether bonds (C–O–C), form CNTs under the catalytic action of Fe. The specific steps are illustrated in Figure 10.

## 4. Conclusions

The alkaline K-catalyzed pyrolytic generation of CNTs from coal is significantly influenced by the pyrolysis temperature, which affects the catalytic properties of KOH and K_2_CO_3_. Below 890 °C, CNTs are not formed in the pyrolysis products. However, numerous CNTs are generated after pyrolysis at 900 °C, with the RC-K-900 and RC-2K-900 samples achieving CNT contents of 9.43 and 8.98 wt.%, respectively. Both samples contained the same amount of K, indicating that KOH is more conducive to the formation of CNTs than K_2_CO_3_. In the K-catalyzed coal pyrolysis process, groups such as C–C, C–H, and C–O–C act as carbon sources for the generation and growth of CNTs. XRD analysis of the RC-K-900 and RC-2K-900 samples reveals the appearance of the graphite (002) diffraction peak, a significant increase in the L_c_ (002) stacking height, and a significant decrease in the interlayer spacing d_002_. This is ascribed to the production of CNTs in these samples, while the samples pyrolyzed at lower temperatures do not show the formation of CNT. The enhanced G-bands in the Raman spectra of these two samples further confirm the presence of CNTs. The primary catalyst for CNT formation is the inherent Fe in the coal, which predominantly exists as Fe_3_C within the CNTs. The mechanism of CNT generation via K-catalyzed coal pyrolysis is as follows: KOH first reacts with carbon-containing groups in coal (such as ethyl groups) and gets transformed into K_2_CO_3_ and K_2_O. Further, K_2_CO_3_ decomposes at high temperatures and is transformed into K_2_O. The K_2_O derived from KOH or K_2_CO_3_ reacts with aromatic C–C and C–H groups in the coal to form K, which then reacts with the C–O–C groups in the coal to produce the carbon source required for the growth of CNTs. This carbon source ultimately forms CNTs under the action of the Fe catalyst.

## Figures and Tables

**Table 1:** Proximate and ultimate analyses of the sample.

Samples	Proximate Analyses (w_ad_/%)			Ultimate Analyses (w_daf_/%)				
	Moisture	Ash	Volatile Matter	C	H	S	N	O *
RC (raw coal)	5.31	5.75	30.13	83.11	4.81	0.22	0.73	11.13
RC-K-900	1.28	10.86	5.49	85.64	0.33	0.13	0.41	13.49
RC-2K-900	1.36	11.07	5.76	85.19	0.37	0.15	0.38	13.91

ad, air dry basis; daf, dry ash free basis; *, by difference.

**Table 2:** Sample nomenclature.

Samples	600 °C	700 °C	800 °C	900 °C
RC	RC-600	RC-700	RC-800	RC-900
RC-K	RC-K-600	RC-K-700	RC-K-800	RC-K-900
RC-2K	RC-2K-600	RC-2K-700	RC-2K-800	RC-2K-900

**Table 3:** Attribution of functional groups in FTIR analysis [46,48,49,50,51,52,53,54].

Wavenumber (cm^−1^)	Peak Assignment
3440	O–H stretching vibrations
2914	–CH_2_ stretching vibration of aliphatic
2853	–CH_3_ stretching vibration of aliphatic
1590	C=C stretching vibrations of aromatic rings
1060	C–C, C–H stretching vibration of aromatic rings, C–O–C stretching vibration
874, 805, 740	C–H stretching vibration of aromatic rings
590	Si–O stretching vibrations, Si–O–Al compounded stretching vibrations, quartz

## Data Availability

The original contributions presented in this study are included in the article. Further inquiries can be directed to the corresponding author.

## References

[1] Xie Y. (2025). Enhanced mechanical and thermal performance of high-strength engineered geopolymer composites reinforced by hybrid polyethylene fibres and carbon nanotubes. Constr. Build. Mater..

[2] Mousavi S.R., Estaji S., Kiaei H., Mansourian-Tabaei M., Nouranian S., Jafari S.H., Ruckdäschel H., Arjmand M., Khonakdar H.A. (2022). A review of electrical and thermal conductivities of epoxy resin systems reinforced with carbon nanotubes and graphene-based nanoparticles. Polym. Test..

[3] Soni S.K., Thomas B., Kar V.R. (2020). A Comprehensive Review on CNTs and CNT-Reinforced Composites: Syntheses, Characteristics and Applications. Mater. Today Commun..

[4] Deng Y., Zhou G., Miao R., Deng J., Wang L., Shao Q., Shao C. (2025). The electronic transport characteristics subsequent to linear doping with nitrogen or boron in (8,0) single-walled carbon nanotubes. Mater. Sci. Semicond. Process..

[5] Jiang N., Shao Y., Zhao X., Zhang Y., Lu P., Ye L., Yang Q., Qiu J. (2025). Two-dimensional carbon-based nanomaterials convoying dendrite-free Zn/Li metal batteries. Chem. Eng. Sci..

[6] Lawal A.T. (2025). Recent application of carbon nanotubes in energy storage and conversion devices. Carbon Trends.

[7] Li C., Zhong J., Sun Y. (2025). Novel approach to enhance the damping performance of cement-based materials through polymer/carbon nanotube composite coating and gradation of aggregates. Constr. Build. Mater..

[8] Anzar N., Hasan R., Tyagi M., Yadav N., Narang J. (2020). Carbon nanotube—A review on Synthesis, Properties and plethora of applications in the field of biomedical science. Sensors Int..

[9] Arora N., Sharma N.N. (2014). Arc discharge synthesis of carbon nanotubes: Comprehensive review. Diamond Relat. Mater..

[10] Pant M., Singh R., Negi P., Tiwari K., Singh Y. (2021). A comprehensive review on carbon nano-tube synthesis using chemical vapor deposition. Mater. Today Proc..

[11] Kwon O., Park J., Park Y.T. (2024). Current Progress of Carbon Nanotubes Applied to Proton Exchange Membrane Fuel Cells: A Comprehensive Review. Int. J. Precis. Eng. Manuf. Green Technol..

[12] Shifa M., Toor Z.S., Tariq F. (2024). Arc Discharge Synthesis and Multistep Purification of Multiwall Carbon Nanotubes. Nano.

[13] Zhou G., Wu H., Deng Y., Miao R., Lai D., Deng J., Zhang J., Chen Q., Shao Q., Shao C. (2024). Synthesis of high-quality multi-walled carbon nanotubes by arc discharge in nitrogen atmosphere. Vacuum.

[14] Rathinavel S., Priyadharshini K., Panda D. (2021). A review on carbon nanotube: An overview of synthesis, properties, functionalization, characterization, and the application. Mater. Sci. Eng. B.

[15] Nelson P.F. (2023). Environmental Issues: Emissions, Pollution Control, Assessment, and Management.

[16] Zhao Y., Mu W., Yang J., Li G., Zhang H., Wang Y. (2025). Synergistic optimization and control of coal-based impregnating pitch performance by refining and thermal modification. J. Ind. Eng. Chem..

[17] Wu X., Liu J., Wu C., Wu W., Wang Y., Zhao Y., Li G., Zhang G. (2024). Green synthesis of nitrogen and oxygen enriched porous carbon from lignite humate for high-performance supercapacitors. J. Energy Storage.

[18] Gasparotto J., Da Boit Martinello K. (2021). Coal as an energy source and its impacts on human health. Energy Geosci..

[19] Alhassan A., Ozturk I., Al-Zyoud M.F., Bekun F.V. (2024). Coal consumption-environmental sustainability nexus in developed and developing major coal-consuming economies. Heliyon.

[20] Muhammad S., Li Y., Yang H., Jin L., Li D., Hu H. (2025). Removal of elemental mercury from coal combustion flue gas by sodium halides impregnated red mud. J. Fuel Chem. Technol..

[21] Zhang H., Zhu L., Pan Z., Cui J., Wang B., Zhang D., Guo Y., Cheng F. (2025). Exploring the mechanisms of enhanced activated carbon’s toluene adsorption and regeneration by utilizing inherent pyrite in coal. Fuel.

[22] Pang L.S., Wilson M.A. (1993). Nanotubes from coal. Energy Fuels.

[23] Williams K.A., Tachibana M., Allen J.L., Grigorian L., Cheng S.C., Fang S.L., Sumanasekera G.U., Loper A.L., Williams J.H., Eklund P.C. (1999). Single-wall carbon nanotubes from coal. Chem. Phys. Lett..

[24] Qiu J.S., Zhang F., Zhou Y., Han H.M., Hu D.S., Tsang S.C., Harris P.J.F. (2002). Carbon nanomaterials from eleven caking coals. Fuel.

[25] Wang Z., Zhao Z., Qiu J. (2006). Synthesis of branched carbon nanotubes from coal. Carbon.

[26] Qiu J., Wang Z., Zhao Z., Wang T. (2007). Synthesis of double-walled carbon nanotubes from coal in hydrogen-free atmosphere. Fuel.

[27] Awasthi S., Awasthi K., Ghosh A.K., Srivastava S.K., Srivastava O.N. (2015). Formation of single and multi-walled carbon nanotubes and graphene from Indian bituminous coal. Fuel.

[28] Wilson M.A., Patney H.K., Kalman J. (2002). New developments in the formation of nanotubes from coal. Fuel.

[29] Zhang T., Wang Q., Li G., Zhao Y., Lv X., Luo Y., Zhang Y. (2019). Formation of carbon nanotubes from potassium catalyzed pyrolysis of bituminous coal. Fuel.

[30] Das T., Saikia B.K., Baruah B.P. (2016). Formation of carbon nano-balls and carbon nano-tubes from northeast Indian Tertiary coal: Value added products from low grade coal. Gondwana Res..

[31] Zhang T., Wang Q., Lv X., Luo Y., Zhang Y. (2020). Transformation of primary siderite during coal catalytic pyrolysis and its effects on the growth of carbon nanotubes. Fuel Process. Technol..

[32] Yuan J., Wang Y., Tang M., Hao X., Liu J., Zhang G., Zhang Y. (2023). Preparation of N, O co-doped carbon nanotubes and activated carbon composites with hierarchical porous structure for CO2 adsorption by coal pyrolysis. Fuel.

[33] Zhang C., Wang Y., Zhao Y., Zhang G. (2025). Ultrahigh-capacity adsorption of Rhodamine B by N, S co-doped carbon nanotube composites derived from coal. Sep. Purif. Technol..

[34] Tao Z., Zhao Y., Wang Y., Zhang G. (2024). Recent advances in carbon nanotube technology: Bridging the gap from fundamental science to wide applications. J. Carbon Res..

[35] Rashidi A., Akbarnejad M., Khodadadi A., Mortazavi Y., Ahmadpourd A. (2007). Single-wall carbon nanotubes synthesized using organic additives to Co–Mo catalystssupported on nanoporous MgO. Nanotechnology.

[36] Li R., Antunes E.F., Kalfon-Cohen E., Kudo A., Acauan L., Yang W.C.D., Wang C., Cui K., Liotta A.H., Rajan A.G. (2019). Low-temperature growth of carbon nanotubes catalyzed by sodium-based ingredients. Angew. Chem. Int. Ed..

[37] Modekwe H.U., Moothi K., Daramola M.O., Mamo M.A. (2022). Corn cob char as catalyst support for developing carbon nanotubes from waste polypropylene plastics: Comparison of activation techniques. Polymers.

[38] Zhao C., Ge L., Li X., Zuo M., Xu C., Chen S., Li Q., Wang Y., Xu C. (2023). Effects of the carbonization temperature and intermediate cooling mode on the properties of coal-based activated carbon. Energy.

[39] Gabal M., Hoff D., Kasper G. (2007). Influence of the atmosphere on the thermal decomposition kinetics of the CaCO_3_ content of PFBC coal flying ash. J. Therm. Anal. Calorim..

[40] Niu J., Luo L., Cui J., Zhang H., Guo Y., Li L., Cheng F. (2023). Impact of inherent calcium in coal on the structure and performance of activated carbon in flue gas activation: The enhanced mechanism of calcite on the methylene blue adsorption. J. Clean. Prod..

[41] Zhang Y., Jia J., Sun Y., Xu B., Jiang Z., Qu X., Zhang C. (2023). An effective strategy to synthesize well-designed activated carbon derived from coal-based carbon dots via oxidation before activation with a low koh content as supercapacitor electrodes. Nanomaterials.

[42] Hui T.S., Zaini M.A.A. (2015). Potassium hydroxide activation of activated carbon: A commentary. Carbon Lett..

[43] Wang S., Nam H., Nam H. (2020). Preparation of activated carbon from peanut shell with KOH activation and its application for H_2_S adsorption in confined space. J. Environ. Chem. Eng..

[44] Chen W., Gong M., Li K., Xia M., Chen Z., Xiao H., Fang Y., Chen Y., Yang H., Chen H. (2020). Insight into KOH activation mechanism during biomass pyrolysis: Chemical reactions between O-containing groups and KOH. Appl. Energy.

[45] Oginni O., Singh K., Oporto G., Dawson-Andoh B., McDonald L., Sabolsky E. (2019). Influence of one-step and two-step KOH activation on activated carbon characteristics. Bioresour. Technol. Rep..

[46] Okolo G.N., Neomagus H.W., Everson R.C., Roberts M.J., Bunt J.R., Sakurovs R., Mathews J.P. (2015). Chemical–structural properties of South African bituminous coals: Insights from wide angle XRD–carbon fraction analysis, ATR–FTIR, solid state 13C NMR, and HRTEM techniques. Fuel.

[47] Saikia B.K., Boruah R.K., Gogoi P.K. (2007). FT-IR and XRD analysis of coal from Makum coalfield of Assam. J. Earth Syst. Sci..

[48] Wang Q., Zhang T.K., Zhao Y.Q., He S.Q., Zhang Y.F. (2019). Structural evolution and formation mechanisms of caking components of modified lignite in subcritical H_2_O-CO systems. Energy Fuels.

[49] Kotov N., Keskitalo M.M., Johnson C.M. (2025). Nano FTIR spectroscopy of liquid water in the –OH stretching region. Spectrochim. Acta A Mol. Biomol. Spectrosc..

[50] Suhasaria A., Senapati R., Rout P.K., Sengupta S., Ghosal S., Banerjee P., Mukherjee D., Dey S., Sukul D. (2024). Electrochemical behavior of mild steel coated with long alkyl-chain benzothiazole derivatives in acid solution: Effect of aliphatic chain length. Colloids Surf. A.

[51] Khairul W.M., Tagiling A.A., Muzaman A.Q., Rahamathullah R., Mohammed M., Saidin S., Arshad S., Razak I.A., Razak F.I.A., Sapari S. (2025). The experimental and DFT approaches on electronic, thermal and conductivity properties of non-linear optical bearing fused aromatic chalcones towards prospective OLEDs. J. Mol. Struct..

[52] Fathurrahman N.A., Alhaboudal M.A., Mohammed S., Bello A.K., Al-Saadi A.A. (2024). The role of hydrogen bonding in the conformational stability of 2-methoxyresorcinol: Insights from theoretical calculations, SERS spectroscopy, and solvent effect. J. Mol. Liq..

[53] Islam Touahria Y., Chafai N., Moumeni O., Boublia A., Mehri M., Benguerba Y. (2024). Synthesis, characterization, and comprehensive computational analysis of aromatic hydrazone compounds: Unveiling quantum parameters, evaluating antioxidant activity, and investigating molecular docking interactions. J. Mol. Liq..

[54] Sinha A., Wei J. (2023). Phase evolution and mechanical-hydroscopic properties of alkali-silica reaction gels modified by magnesium nitrate. Cem. Concr. Compos..

[55] Spencer W., Senanayake G., Altarawneh M., Ibana D., Nikoloski A.N. (2024). Review of the effects of coal properties and activation parameters on activated carbon production and quality. Miner. Eng..

[56] Kim J.H., Lee G., Park J.E., Kim S.H. (2021). Limitation of K_2_CO_3_ as a chemical agent for upgrading activated carbon. Processes.

[57] Li Y., Zhu T., Ling Y., Yin Y., Nong G. (2024). Solid waste of calcium lignin replaces fossil fuel power by gasification to reduce CO_2_ emissions. Process Saf. Environ. Prot..

[58] Wang Y., Yao S., Zheng H., Zuo Z., Liu Y. (2025). Nanomechanical properties of anthracite and graphite: The role of heteroatom functional groups and structural evolution. Int. J. Coal Geol..

[59] Xiong Z., Syed-Hassan S.S.A., Hu X., Guo J., Chen Y., Liu Q., Wang Y., Su S., Hu S., Xiang J. (2018). Effects of the component interaction on the formation of aromatic structures during the pyrolysis of bio-oil at various temperatures and heating rates. Fuel.

[60] Behera S. (2008). Carbon: The Material and Its Characterization by Raman Spectroscopy.

[61] Sparavigna A.C. (2024). Graphene, Graphene Oxide and Carbon Nanotubes in Raman Spectroscopy. Int. J. Sci..

[62] Li J., Lan H., Liu H., Zhang G., An X., Liu R., Qu J. (2019). Intercalation of nanosized Fe3C in iron/carbon to construct multifunctional interface with reduction, catalysis, corrosion resistance, and immobilization capabilities. ACS Appl. Mater. Interfaces.

[63] Jiang W., Gu L., Li L., Zhang Y., Zhang X., Zhang L., Wang J., Hu J., Wei Z., Wan L. (2016). Understanding the high activity of Fe-N-C electrocatalysts in oxygen reduction: Fe/Fe_3_C nanoparticles boost the activity of Fe-Nx. J. Am. Chem. Soc..

[64] Strydom C., Collins A., Bunt J. (2015). The influence of various potassium compound additions on the plasticity of a high-swelling South African coal under pyrolyzing conditions. J. Anal. Appl. Pyrolysis.

[65] Sergeev D., Yazhenskikh E., Kobertz D., Müller M. (2019). Vaporization behavior of Na_2_CO_3_ and K_2_CO_3_. Calphad.

[66] Tachimoto H., Abe I. (2002). Activated Carbon Application Technology—Its Maintenance Management and Problems.

